# A case report of neuralgic amyotrophy

**DOI:** 10.3389/fneur.2024.1392766

**Published:** 2024-05-23

**Authors:** Jianfeng Sun, Ji Mao, Fengfei Zhang, Xi Gui, Junfeng Liao

**Affiliations:** ^1^Department of Rehabilitation Medicine, Southern Theater Command General Hospital of the People's Liberation Army, Guangzhou, China; ^2^Department of Ultrasonography, Southern Theater Command General Hospital of the People's Liberation Army, Guangzhou, China

**Keywords:** neuralgic amyotrophy, high-frequency ultrasound, differential diagnoses, diagnosis, precision therapy

## Abstract

Neuralgic muscular atrophy is not uncommon in clinical practice. Due to the different branches of brachial plexus involved in the lesion, the clinical symptoms are different, and there is a lack of clear imaging diagnostic criteria, so the diagnosis of this disease brings great challenges to clinicians. We have certain experience in the diagnosis and treatment of this disease, and hereby select a representative case of neuralgic muscular atrophy to share its diagnosis and treatment process, focusing on analyzing the characteristic symptoms of this disease, valuable imaging data and targeted treatment, so as to enable clinicians to better understand this disease.

## 1 Introduction

Neuralgic amyotrophy (NA), also referred to as idiopathic brachial plexitis and Parsonage-Turner syndrome, is a peripheral nerve disorder characterized by the sudden onset of acute severe pain in one or both shoulders and the rapid onset of weakness in the muscles of the shoulder girdle and upper arm. The minimum annual incidence of NA is two to three per 100,000, but this might be an underestimation, the annual incidence could be at least 20–30 cases per 100,000 individuals. Contrary to initial perceptions of its rarity, NA is not an uncommon ailment. Patients with NA generally have a good prognosis, with previous studies reporting that 80–90% of patients recovered within 2–3 years after the onset (1). However, some recent studies reported that only 4.1% of NA patients showed a total recovery from paresis (2).

Neuralgic amyotrophy is assumed to have a complex aetiology, in which autoimmune, inflammatory, mechanical and genetic factors appear to play a role (3). Our clinical observations have identified a subset of patients with a history of viral hepatitis, vaccination, previous fracture surgeries, and a history of COVID-19. Conversely, another subset of patients presents without clear precipitating factors or relevant medical history.

NA is a diagnosis of exclusion. Currently, no standardized morphologic confirmatory test for NA has been proposed. There is no laboratory test to diagnose the condition. It is diagnosed with the help of history, physical exam, magnetic resonance neurography (MRN), musculoskeletal ultrasound, and EMG studies. While NA is incompletely understood and often difficult to diagnose, early recognition may prevent unnecessary tests and interventions and in some situations, allow for prompt treatment, which can potentially minimize adverse long-term sequalae (4).

We present a case of neurotrophic amyotrophy in a middle-aged male patient. Early diagnosis and prompt treatment yielded favorable outcomes. The comprehensive medical history is detailed below.

## 2 Case report

Patient is a middle-aged male with an acute onset of symptoms. He was admitted due to “left neck and shoulder pain for 3 weeks.” The patient reported sudden onset of left neck and shoulder pain on the night of October 2, 2022, with significant pain in the left scapular and lateral shoulder areas, extending to the left neck. The pain was described as throbbing, severe, and intolerable, significantly impacting daily life and work. Symptoms worsened in the supine position, preventing sleep, but were slightly relieved when sitting or standing. There was no fever, headache, numbness in the limbs, or shoulder joint mobility impairment. Oral nonsteroidal anti-inflammatory drugs were ineffective. Temporary relief was noted during cervical traction, but pain recurred after traction cessation. Cervical spine MRI revealed protrusion of the C4/5 and C5/6 intervertebral discs. Magnetic resonance imaging of the left shoulder joint showed no significant abnormalities. Considering possible compression of the suprascapular nerve, a left suprascapular nerve block (scapular notch) was administered, resulting in temporary pain relief on the day of treatment but recurrence the next day. Subsequent consultations with orthopedics and neurology suggested intervertebral disc protrusion with nerve root involvement. Treatment with mannitol and dexamethasone injections provided limited relief. The patient was then admitted to the Rehabilitation Medicine Department. The pain persisted in the left scapular and lateral shoulder areas, with a Visual Analog Scale (VAS) score of 9. The diagnosis upon admission was neuralgic amyotrophy. The patient received suprascapular nerve blocks (origin of the upper trunk and scapular notch) and brachial nerve blocks (quadrilateral space), along with oral cobamamide tablets and pregabalin capsules. Three weeks after the onset, the patient exhibited a decrease in strength in left shoulder joint abduction and external rotation. By the fifth week, pain gradually diminished, and muscle strength further declined. At 8 weeks post-onset, pain significantly reduced with a VAS score of 2, not affecting sleep or work, but the patient still experienced left upper limb weakness. Throughout the course, the patient maintained general mental well-being, fair appetite, poor sleep, and regular bowel and bladder habits. No history of allergies to food or medication, recent vaccination, hepatitis, surgical procedures, or family history of similar disorders was reported.

## 3 Investigation

### 3.1 Physical examination

**Figure 1:**
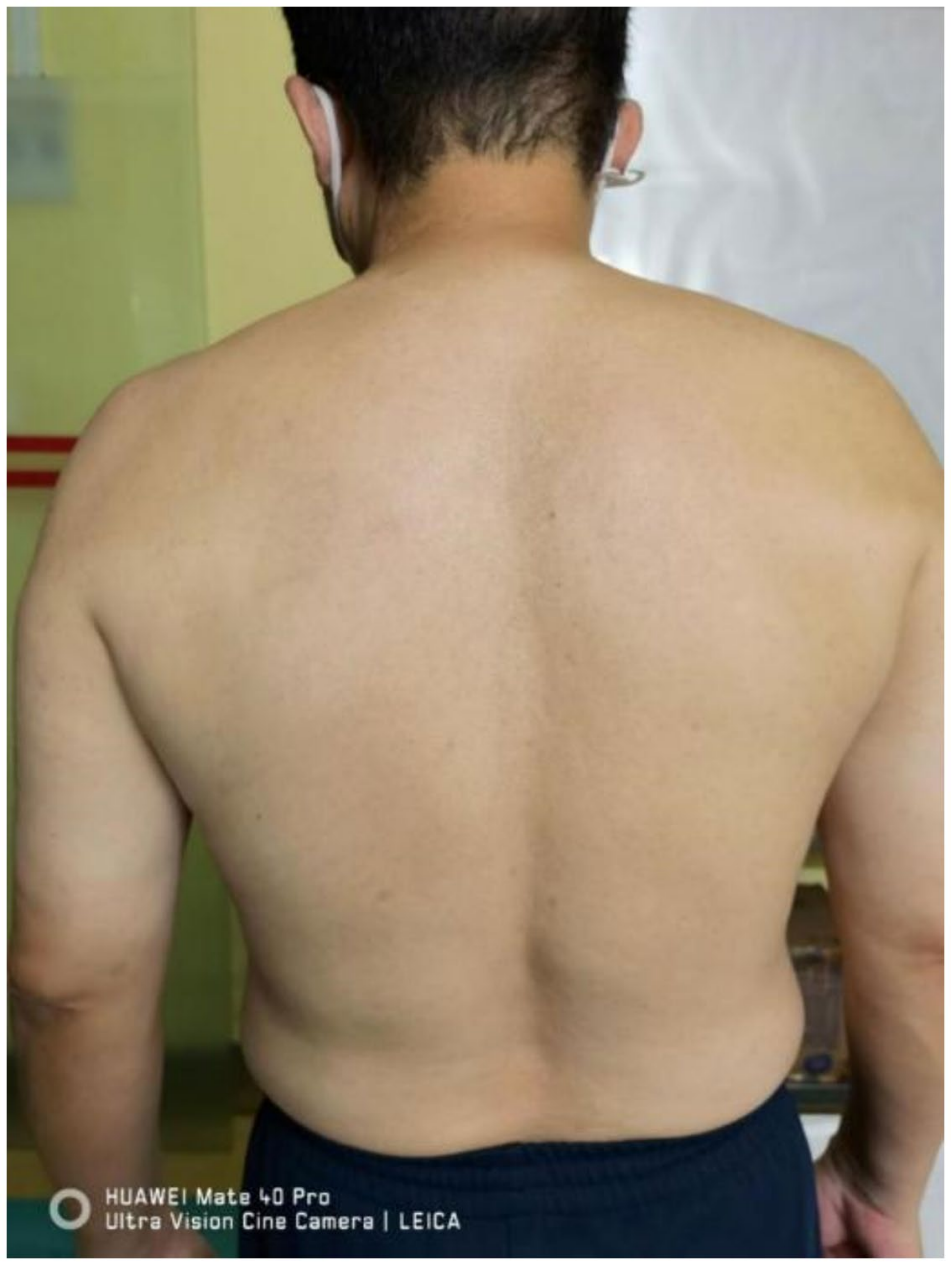
Atrophy of muscles in the left scapular region.

Vital signs stable, walked into the department. Cooperative during examination. No abnormality in the range of motion of the neck and shoulder joints. Atrophy of the left supraspinatus and infraspinatus muscles noted (Figure 1). Tenderness over the left C4-6 facet joints (+), tenderness over the left middle scalene muscle (+), positive result in the neck separation test, negative left intervertebral foramen compression test, negative left brachial plexus tension test, negative left intervertebral foramen compression test, negative Lhermitte’s sign, negative pain arc test. Left shoulder abduction strength rated at 4, left shoulder external rotation strength rated at 4. No abnormalities observed in the strength of left elbow flexion, extension, or grip. No abnormalities in the sensation of the left scapular area and upper limb. Bilateral biceps tendon reflex (++), bilateral triceps tendon reflex (++), negative Hoffmann’s sign. Visual Analog Scale (VAS) score: 9.

### 3.2 Imaging studies

#### 3.2.1 Ultrasound (October 24, 2022, November 18, 2022)

**Figure 2:**
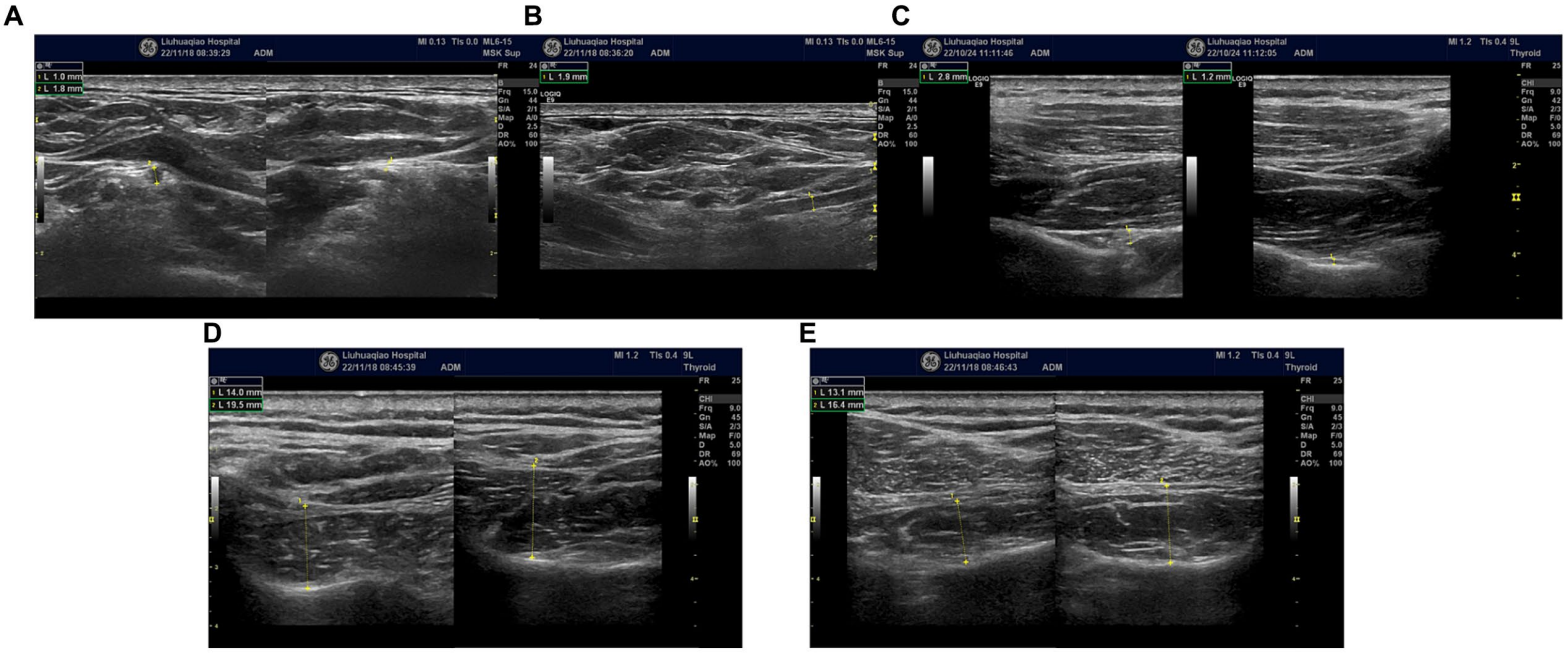
**(A)** Bilateral suprascapular nerve origins; swelling of the left suprascapular nerve. Left side, had a diameter (short axis) of 1.8 mm, while on the right side it measured 1 mm. **(B)** Swelling of the left suprascapular nerve at its origin. The diameter (long axis) of nerve was 1.9 mm. **(C)** Bilateral suprascapular notches; swelling of the left suprascapular nerve, the diameters (short axis) were 2.8 mm on the left side and 1.2 mm on the right side. **(D)** Bilateral comparison of the supraspinatus muscles; atrophy of the left supraspinatus muscle (short axis). **(E)** Bilateral comparison of the infraspinatus muscles; atrophy of the left infraspinatus muscle (short axis).

Swelling of the left suprascapular nerve. The suprascapular nerve at its origin, left side, had a diameter (short axis) of 1.8 mm, while on the right side it measured 1 mm (Figure 2A). The diameter (long axis) of the left suprascapular nerve at its origin was 1.9 mm (Figure 2B). At the suprascapular notch, the diameters (short axis) were 2.8 mm on the left side and 1.2 mm on the right side (Figure 2C). Additionally, there was observed atrophy of the left supraspinatus and infraspinatus muscles (Figures 2D,E). The brachial plexus in the supraclavicular fossa showed no significant abnormalities, and no significant atrophy of the deltoid muscle.

#### 3.2.2 Nerve conduction studies (October 27, 2022)

**Figure 3:**
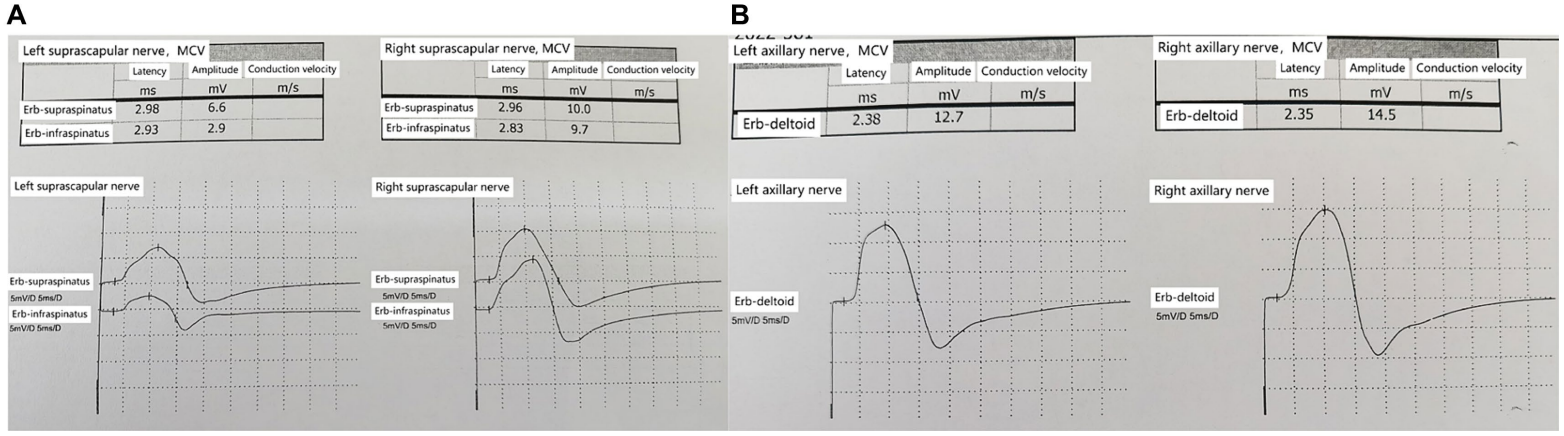
**(A)** Bilateral suprascapular nerves comparison; reduced amplitude of motor action potentials in the left suprascapular nerve. **(B)** Bilateral axillary nerves comparison; reduced amplitude of motor action potentials in the left axillary nerve.

Injury to the left suprascapular nerve and axillary nerve, with reduced amplitude of motor action potentials compared to the right side, within normal latency ranges (Figures 3A,B).

#### 3.2.3 Cervical spine MRI (October 8, 2022)

**Figure 4:**
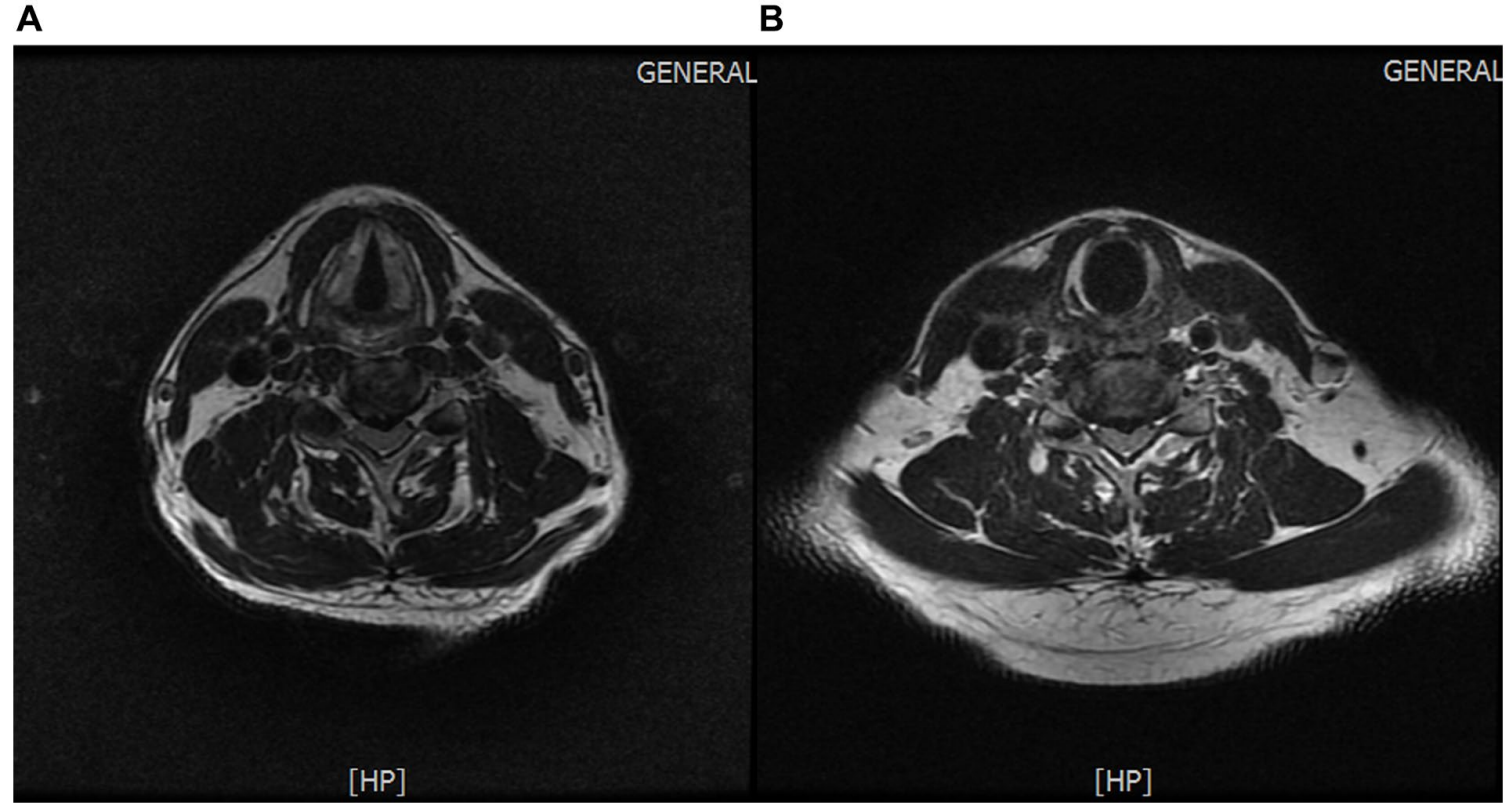
**(A)** Protrusion of the C4/5 intervertebral disc. **(B)** Protrusion of the C5/6 intervertebral disc.

Protrusion of the C4/5 and C5/6 intervertebral discs (central type) (Figures 4A,B).

## 4 Treatment

The patient underwent ultrasound-guided nerve block treatment, receiving injections of Compound Betamethasone Injection 1 mL (Dexamethasone disodium phosphate 2 mg and betamethasone 5 mg) Lidocaine Hydrochloride Injection 80 mg, and 0.9% Sodium Chloride Injection 15 mL. The injections were targeted at the suprascapular nerve at the origin of the upper trunk, scapular notch, and quadrilateral space of the axillary nerve, with a total of 5 nerve block treatments administered at a frequency of once per week. Simultaneously, the patient was prescribed Pregabalin Capsules 150 mg orally twice daily, to be taken for 6 weeks and gradually tapered off. Cobamamide Tablets 0.5 mg were prescribed orally three times daily, to be taken for 8 weeks. The oral medications were discontinued by the 11th week of onset, and the pain gradually subsided. Some discomfort in the neck and shoulder was noted during certain movements, with a Visual Analog Scale (VAS) score of 2. The pain did not affect daily activities or work, but there was still no recovery in left shoulder abduction and external rotation strength. At 3 months post-onset, occasional pain in the left shoulder was reported, with a noticeable decrease in frequency compared to before. Left shoulder strength was similar to previous assessments. By 5 months post-onset, the pain had largely disappeared, and left shoulder strength began to recover, although not yet to normal levels.

## 5 Discussion

Diagnosis of NA poses significant challenges, especially in its early stages, and the current situation may be attributed to several factors. Firstly, there is a lack of awareness among clinical practitioners regarding this condition. Often, when faced with such diseases, they are prone to misdiagnose it as other conditions such as cervical radiculopathy or peripheral nerve entrapment. Secondly, the clinical manifestations of NA are complex and variable, with involvement of non-fixed nerves. NA can affect almost every brachial plexus nerve, but most frequently involvement of the upper trunk. The nerves most frequently affected by NA are the suprascapular, long thoracic, median and anterior interosseous nerve (AIN) branch, radial and posterior interosseous nerve (PIN) branch, axillary, spinal accessory, and musculocutaneous (4). Moreover, the nerves affected by NA vary at different stages of the disease. In the early stages, symptoms may manifest as suprascapular nerve involvement, and as the condition progresses, there may be involvement of the anterior interosseous nerve and sympathetic nerves, leading to symptoms such as nail nutritional disorders and skin eczematous changes. It has been reported that NA not only affects brachial plexus nerves but also cranial nerves, including the accessory nerve, hypoglossal nerve, glossopharyngeal nerve, and even the recurrent laryngeal nerve and nerves in the lower limbs, making the clinical presentation highly complex and variable. Furthermore, the absence of objective imaging diagnostic criteria adds to the diagnostic challenge. Although cervical spine MRI in patients often reveals radiological features of disc herniation, without strict adherence to precise localization diagnostics, there is a high risk of misdiagnosing it as cervical radiculopathy. Clinical physicians must rigorously perform physical examinations and combine qualitative and localization diagnostics to accurately diagnose this condition. The author, having previous experience in diagnosing and treating such diseases, was able to make an early diagnosis in this patient who had previously received inadequate treatment from neurology and spine surgery departments. Prior to diagnosing neuropathic amyotrophy, necessary clinical differentials were thoroughly evaluated.

### 5.1 Differential diagnosis from cervical radiculopathy

The patient sought consultation from the spine surgery department upon onset of symptoms. Given the pain in the scapular and lateral shoulder region, coupled with cervical spine MRI findings indicating disc protrusion at C4/5 and C5/6, along with corresponding ligamentum flavum hypertrophy and spinal canal stenosis (minimum anterior–posterior diameter of 5.5 mm) causing spinal cord compression with uniform signal intensity, the spine surgeon diagnosed the condition as cervical radiculopathy. Initial treatment with oral and intravenous drip medications yielded unsatisfactory results. The disease presented with typical neuropathic pain and abnormal cervical magnetic resonance imaging (MRI) results, leading to a misdiagnosis as cervical radiculopathy. The patient’s MRI revealed disc protrusion at C4/5, potentially compressing the C5 nerve root, leading to clinical manifestations such as weakened deltoid and supraspinatus muscle strength and shoulder pain. In careful analysis of the patient’s symptoms, physical examination, and imaging studies, several points inconsistent with cervical radiculopathy became evident. Firstly, the intensity of the patient’s pain was severe (Visual Analog Scale score: 9), which is atypical for cervical radiculopathy, where such intense pain is uncommon. Additionally, the pain did not alleviate in the supine position with external support to the head and neck, contrary to the usual relief seen in cervical radiculopathy. Furthermore, the exacerbation of symptoms in the standing position with neck support contradicts the typical characteristics of cervical radiculopathy. Secondly, upon physical examination analysis, the weakness was observed in the left supraspinatus, infraspinatus, and deltoid muscles, while biceps brachii muscle strength on the left side remained unaffected. Sensory examination revealed no abnormalities on the left lateral upper arm. Reflex examination showed no abnormalities in the left biceps tendon reflex. Additional clinical signs, such as negative left brachial plexus traction test and left intervertebral foramen compression test, did not align with the clinical presentation of cervical radiculopathy affecting C5 or C6 nerve roots. Finally, upon MRI analysis (Figures 4A,B), although the patient’s cervical spine MRI exhibited multilevel disc protrusion, the severity at C4/5 and C5/6, both centrally protruding, did not show signs of left nerve root compression. Furthermore, ultrasound examination did not reveal swelling of the left C5 and C6 nerve roots. Through a comprehensive evaluation and analysis of symptoms, physical signs, and imaging studies, a three-step diagnostic process was employed to rule out the possibility of cervical radiculopathy.

### 5.2 Differential diagnosis from suprascapular nerve compression syndromes

In the early stages of the disease, the possibility of suprascapular nerve compression was considered in this patient. The patient presented with pain in the supraspinatus, infraspinatus, and the lateral aspect of the shoulder joint. The suprascapular nerve innervates the supraspinatus and infraspinatus muscles and sends branches to the shoulder joint capsule. Ultrasound examination revealed swelling of the suprascapular nerve at the suprascapular notch on the left side. Ultrasound-guided nerve blockade at this site provided pain relief for 2 days, confirming compression of the suprascapular nerve at this location. High-frequency ultrasound examination at the junction of the clavicle and the midline of the clavicle revealed swelling of the suprascapular nerve below the belly of the omohyoid muscle in the short-axis view (Figure 2A). In the long-axis view (Figure 2B) obtained by rotating the probe 90 degrees at the site of swelling, the suprascapular nerve was observed to be swollen, although “hourglass constriction” was not evident. Short-axis scanning at the suprascapular notch continued to reveal nerve swelling, indicating that the suprascapular nerve extends from the brachial plexus upper trunk to the suprascapular notch, and the nerve is in a swollen state at this location. Compression of the suprascapular nerve commonly occurs at the suprascapular and spinoglenoid notches, and nerve swelling may occur at the compressed site, while the nerve’s morphology remains normal in the non-compressed area. This is a key point in differentiating neuropathic amyotrophy from suprascapular nerve compression. If a single nerve shows swelling in different areas, it can be distinguished from peripheral nerve compression diseases and can serve as an imaging sign for diagnosing neuropathic amyotrophy. Currently, some scholars propose “hourglass constriction” as an imaging diagnostic criterion under ultrasound for this condition. However, in the early stages of the disease, nerve swelling is the primary manifestation, and the typical “hourglass constriction” sign may not be present. Some patients do not exhibit “hourglass constriction” changes throughout the course of the disease. Nerve conduction study results indicated damage to the left suprascapular nerve and axillary nerve, with less reduction in amplitude of the left axillary nerve motor wave (Figures 3A,B). Consequently, there was atrophy of the left supraspinatus and infraspinatus muscles (Figures 2D,E), while atrophy of the deltoid muscle was less pronounced. Ultrasound examination revealed no significant swelling of the axillary nerve within the quadrilateral space. Based on these findings, it is inferred that inflammation of the suprascapular nerve is the primary pathology in this case, simultaneously affecting the axillary nerve. The suprascapular nerve originates from the upper trunk of the brachial plexus, while the axillary nerve arises from the posterior cord of the brachial plexus. Clinically, it is improbable for compression at one site to simultaneously affect these two nerves, whereas neuropathic amyotrophy can explain inflammation of different nerves. Current literature reports suggest that isolated suprascapular nerve compression is not common, and swelling of the suprascapular nerve is more often a form of neuropathic amyotrophy. This perspective aligns with the presentation of this patient.

### 5.3 Role of investigations in the diagnosis

Brachial plexitis is a diagnosis of exclusion. It is diagnosed with the help of history, physical exam, imaging, and EMG studies. There is no laboratory test to diagnose the condition (5). We recommend relying heavily on history and physical examination to determine which nerves are affected and should therefore be assessed with ultrasound (4). We recommend scanning the nerve along its entire course or to the extent of which a clear image can be obtained. Nerve swelling alone is the most common sonographic finding in NA (6). At sites of constriction, slowly rotate the probe 90 degrees with the indicator toward the patient’s head to obtain the long axis view to determine if “hourglass constriction” is present. At a pathologic site, slow dynamic shortaxis scanning should be performed to look for fascicular entwinement (4). While hourglass constrictions and fascicular entwinement are less common, these features are critical to recognize as they may provide insight regarding prognosis and need for intervention beyond conservative measures (4). In addition to the distinctive ultrasonographic features of the aforementioned nerves, some patients may also exhibit swelling of the C5 or C6 nerve roots on ultrasound examination. Alternatively, enlargement of the upper trunk of the brachial plexus may be observed within the interscalene. HRUS is an inexpensive, real-time, point of care modality that identifies findings specific to NA and may help predict prognosis and need for ultrasound-guided procedures or surgical intervention. MRI can now more reliably identify nerve changes in acute, subacute, and chronic phases of NA. However, MRI is time consuming, costly, and lacks sensitivity for identifying detailed nerve structure and pathology (4). In this case, cervical spine magnetic resonance imaging (MRI) plain scans did not reveal compression of the nerve roots, providing crucial evidence for ruling out nerve root-type cervical spondylosis. Electromyography is the best study to demonstrate demyelination and to be performed after 3 weeks of symptoms onset to demonstrate abnormality (5). Nerve conduction studies have played a supportive role in the diagnosis of neuropathic muscular atrophy.

### 5.4 Treatment of neuralgic amyotrophy

NA leaves one-half to two-thirds of patients with disability and pain over the years. Most patients are seen at a late stage, at which time therapy with corticosteroids is generally not considered successful (7, 8). A standard physical therapy approach was ineffective or aggravated symptoms in more than 50% (7). When the patient received scapular notch block therapy following onset, results were suboptimal, likely due to the oversight of swelling at the proximal portion of the upper trunk. Subsequent administration of corticosteroid injections at sites of neural swelling, including the initiation point of the suprascapular nerve and the scapular notch, led to a gradual reduction in pain. Literature suggests that the pathogenesis of neuralgic amyotrophy involves an immune basis, characterized by inflammatory changes driven by immune reactions, rather than nerve entrapment (9). We strongly endorse this pathological mechanism, as it provides a theoretical basis for the use of glucocorticoid therapy in the treatment of this condition. Therefore, early diagnosis and initiation of steroid anti-inflammatory treatment are particularly important. Neuralgic amyotrophy benefits from early intervention with corticosteroid anti-inflammatory treatment guided by clinical symptoms and ultrasound localization of affected nerves. This approach aims to alleviate neural swelling, shorten the disease course, and mitigate the intensity of patient pain, thereby expediting the recovery process. Early anti-inflammatory treatment can also halt further progression of the disease. Patients in the later stages of the disease may exhibit “hourglass constriction” in the nerves, indicating a prolonged course and significant nerve damage. This phenomenon suggests suboptimal treatment efficacy. Therefore, it is crucial to intervene early to prevent disease progression. Concurrently, the use of oral pregabalin capsules complements the treatment by alleviating neuropathic pain, contributing to a more effective pain relief strategy for the patient. Kim JG and Chung SG reported a case of herpetic brachial plexopathy treated with the ultrasound-guided corticosteroid injection (10). We have successfully cured 5 patients clinically through the administration of glucocorticoid injections, precisely following the aforementioned treatment regimen. However, the number of cases remains limited. Presently, there is insufficient evidence to establish the superiority of localized corticosteroid treatment over oral corticosteroid therapy at the site of nerve lesions in neuralgic amyotrophy. Further studies with larger sample sizes are required to substantiate these findings.

## 6 Conclusion

Neuralgic amyotrophy manifests with a complex and variable clinical profile, affecting both the clavicular upper and lower branches of the brachial plexus. Consequently, physicians must possess a profound understanding of brachial plexus anatomy to facilitate early disease diagnosis. Intense pain in the scapular region (VAS≧8) emerges as a pivotal early symptom of neuralgic amyotrophy. Positive physical examination findings in this region can be easily confused with those of cervical radiculopathy, but collaboration with cervical spine MRI can rule out nerve root compression. When considering specific nerve involvement, high-frequency ultrasound is essential to scan the roots, trunks, divisions, and cords of the brachial plexus, exploring the entire nerve pathway from its origin. This aids in distinguishing peripheral nerve compression. Ultrasound examination revealing swelling or “hourglass constriction” in different segments of the same nerve represents the most prevalent ultrasonic imaging in this condition. Nerve conduction studies contribute to further delineate the affected nerves, providing robust support for clinical and ultrasound diagnoses. Hormone injections administered at the swollen sites of the affected nerves significantly alleviate pain and shorten the course of the disease. This case lacks a comparative ultrasound imaging of the affected nerves before and after treatment, and the clinical application of the treatment regimen has been limited in terms of case numbers. Additionally, there is a lack of observation regarding long-term adverse reactions following steroid administration. These aspects will be important considerations for future research. This comprehensive review aims to raise awareness among clinicians, emphasizing the importance of early diagnosis and intervention in the onset of the disease to maximize relief for the afflicted individuals.

## Data availability statement

The original contributions presented in the study are included in the article/supplementary material, further inquiries can be directed to the corresponding author.

## Ethics statement

The studies involving humans were approved by Southern Theater General Hospital Scientific Research Ethics Committee (NZLLK2024010). The studies were conducted in accordance with the local legislation and institutional requirements. Written informed consent for participation was not required from the participants or the participants’ legal guardians/next of kin in accordance with the national legislation and institutional requirements. Written informed consent was obtained from the individual(s) for the publication of any potentially identifiable images or data included in this article.

## Author contributions

JS: Writing – original draft, Data curation, Formal analysis. JM: Writing – original draft, Data curation, Formal analysis. FZ: Writing – original draft, Data curation, Formal analysis. XG: Writing – original draft, Data curation, Formal analysis. JL: Writing – original draft, Data curation, Formal analysis.
